# Suspected oncologic adverse reactions associated with interleukin‐23 inhibitors in EudraVigilance: Comparative study and gender distribution

**DOI:** 10.1002/prp2.1130

**Published:** 2023-08-24

**Authors:** Fabrizio Calapai, Carmen Mannucci, Luigi Cardia, Mariaconcetta Currò, Gioacchino Calapai, Emanuela Esposito, Ilaria Ammendolia

**Affiliations:** ^1^ Department of Clinical and Experimental Medicine University of Messina Messina Italy; ^2^ Department of Chemical, Biological, Pharmaceutical and Environmental Sciences University of Messina Messina Italy; ^3^ Department of Biomedical and Dental Sciences and Morphological and Functional Imaging University of Messina Messina Italy; ^4^ Department of Human Pathology of Adult and Childhood “Gaetano Barresi” University of Messina Messina Italy

**Keywords:** adverse reactions, cancer, guselkumab, IL‐23 inhibitors, pharmacovigilance, psoriasis, real‐world, risankizumab, tildrakizumab

## Abstract

Psoriasis is a chronic inflammatory skin disease characterized by plaque formation. Interleukin (IL)‐23 is upregulated in psoriatic lesions and is thought to be a major regulator of the Th17 pathway in psoriasis pathogenesis. Three monoclonal antibodies targeting the IL‐23p19 subunit, guselkumab, tildrakizumab, and risankizumab, have been approved for psoriasis therapy. The balance between cytokines IL‐23 and IL‐12 can affect antitumor and pro‐tumor immune activities, and patients with psoriasis may have higher rates of cancer than the general population. Moreover, a chronic inflammatory state typical of psoriasis may induce protumorigenic effects, however, the potential risk of malignancy in patients taking these drugs remains largely unknown. This study investigated the occurrence of malignancies as suspected adverse reactions (SARs) potentially associated with IL‐23 inhibitors by analyzing real‐world data from the European EudraVigilance database. Although indicatory, these real‐world data seem to confirm the potential association between the IL‐23 inhibitors risankizumab and tildrakizumab, and the occurrence of SARs linked to cancer in patients with psoriasis and, according to a gender perspective, they show that this relationship is asymmetrically distributed between women and men, with a clear prevalence of oncologic SARs in men.

**Abbreviations**
CI: confidence interval
EEA: European Economic Area
EMA: European Medicines Agency
ICSR: individual case safety report
Ig: immunoglobulin
IL: interleukin
MedDRA: Medical Dictionary of Regulatory Activities
ROR: reporting odds ratio
SAR: suspected adverse reaction

## 1 INTRODUCTION

Psoriasis is a chronic inflammatory skin disease affecting approximately 3% of general population. It is characterized by a thickened and nucleated keratinocyte layer, and erythematous squamous and delimited plaques. Psoriatic lesions are the result of the interactivity of dermal infiltrates of activated dendritic cells, T‐cells, and cytokines, including interferon‐γ, tumor necrosis factor‐α, interleukin (IL)‐12, IL‐17, and IL‐23. IL‐23 is upregulated in psoriatic lesions and is crucial for regulating the Th17 pathway involved in the development of psoriasis.^1^ This cytokine is composed of p19 and p40 subunits binding to the receptor for IL‐23 and IL‐12 receptor b1, causing the triggering of pro‐inflammatory Janus kinase 2, tyrosine kinase 2, and signal transducer and activator of transcription. IL‐23 induces in Th17, formation of IL‐17 from naive T‐cells, and cellular activation by IL‐17 leads to the release of pro‐inflammatory cytokines.^2^ Based on these findings, three monoclonal antibodies having as a target the IL‐23p19 subunit‐guselkumab, tildrakizumab, and risankizumab have been approved. Guselkumab is a human immunoglobulin (Ig) G1 monoclonal antibody against p19 subunit of IL‐23 that inhibits IL‐23 intracellular signaling. Tildrakizumab is a humanized IgG1/x targeting the p19 (80) subunit of IL‐23. Risankizumab is a selective IgG1 binding with high affinity to the IL‐23 subunit p19.^3^ IL‐12 promotes Th1 immunity and IL‐23 stimulates Th17 immunity. Increased preclinical evidence has also shown that the equilibrium between IL‐12 and IL‐23 is critical in tumorigenesis and that impairment of IL‐12 and/or IL‐2 3 signaling both promotes and inhibits tumor growth. Although the mechanisms underlying these biological activities are not fully understood and the interpretation of pre‐clinical studies on the involvement of IL‐12 and IL‐23 in tumor biology is not univocal, clinical studies indicate IL‐23 inhibitors as drugs with a good safety profile. Patients with psoriasis may have higher rates of non‐melanoma skin cancer and other neoplastic diseases than the general population owing to impaired immune surveillance, immunomodulatory therapy, and chronic inflammation.^4^ This can be due to chronic inflammation, distinctive features of psoriasis, and potential pro‐tumorigenic effects. However, the debate on the potential risk of malignancy in patients with psoriasis taking these drugs remains unresolved to a large extent.^5^

The safety and efficacy of guselkumab, risankizumab, and tildrakizumab were investigated in real‐world in an observational, retrospective, multicenter cohort study that analyzed the Psoriasis Registry in Austria (PsoRA). In this study, adverse events observed were mainly non‐severe infections, occurring in 11.7% of patients, showed no main differences among the three IL‐23 inhibitors.^6^ Other evidence obtained through the analysis of real‐world data indicates that IL‐23 inhibitors are generally well tolerated without any emphasis on suspected oncological adverse events.^7^

This study contributes to the literature by investigating the occurrence of malignancies as suspected adverse reactions (SARs) potentially associated with IL‐23 inhibitors and their distribution by sex, by analyzing real‐world data contained in the database EudraVigilance.

## 2 METHODS

EudraVigilance is a database containing SARs linked to drugs licensed for market use in the European Union (EU). The European Medicines Agency (EMA) directs and controls the EudraVigilance. In this data bank, SARs to IL‐23 inhibitors guselkumab, risankizumab, and tildrakizumab are described in individual case safety reports (ICSRs), similar to other drugs. Spontaneous signaling of SARs involves medical events that have been observed following the use of a drug, but are not necessarily caused by the same medicine.^8^ In the EudraVigilance system, all ICSRs are signaled by healthcare or nonhealthcare professionals. For this analysis, only ICSRs reporting SARs signaled by healthcare professionals were retrieved from January 1, 2020 to December 31, 2022. The ICSRs in EudraVigilance are available as cases from the European Economic Area (EEA) countries. Only reports from EEA countries and the United Kingdom (UK) were considered. The public version of the EudraVigilance database was used (http://www.adrreports.eu/en/search.html; accessed March 31, 2023). ICSR‐reporting SARs were selected based on the Medical Dictionary of Regulatory Activities (MedDRA). MedDRA is a standardized, validated medical tool consulted by regulatory authorities and the pharmaceutical industry.^9^ Data on patient characteristics (age group and sex), type of adverse reactions (often more than one for each ICSR), and primary source qualifications were extracted from all ICSRs. According to the International Council on Harmonization E2D guidelines, ICSRs are considered as serious if they are life‐threatening, result in death, cause/prolong hospital recovery, result in disability, are a congenital anomaly/birth defect, or are a medically important condition. Duplicate or incomplete ICSRs were not considered for the analysis.

The primary outcome of interest was the analysis of ICSRs reporting malignancies as SARs related to the IL‐23 inhibitors guselkumab, risankizumab, and tildrakizumab in—3 years 2020–2021–2022. The odds ratio, its 95% confidence interval, and sex difference were calculated using reporting odds ratio (ROR) method.^10^ Descriptive statistical analyses were performed using the statistical software SPSS version 28.0.0.0 (190).

## 3 RESULTS

During the years 2020–2021–2022, spontaneous signaling of 416 cases of suspected SARs were reported in EudraVigilance for IL‐23 inhibitors used in the treatment of psoriasis and arthritic psoriasis. Of these, 53 (12.7%) were related to oncologic diseases. Table 1 lists all oncological diseases signaled as SARs for guselkumab, risankizumab, and tildrakizumab in, years 2020–2021–2022.

**TABLE 1:** Oncologic suspected adverse reactions (SARs) signaled in relationship with prescription of IL‐23 inhibitors guselkumab, risankizumab, and tildrakizumab in psoriatic patients of the European Economic Area and the United Kingdom in the years 2020–2021–2022.

Risankizumab		Guselkumab		Tildrakizumab	
Men	Women	Men	Women	Men	Women
Squamous cell carcinoma of lung	Laryngeal cancer	Brain neoplasm	Endometrial cancer (2)	Renal cell carcinoma	B‐cell lymphoma
Testis cancer	Malignant melanoma (2)	Adenocarcinoma pancreas	Bladder cancer	Seminoma	
Metastases to lymph nodes (2)	Cervix carcinoma	Hepatocellular carcinoma	Breast cancer (2)	Malignant melanoma in situ (2)	
Diffuse large B‐cell lymphoma	Vulval cancer	Gastrointestinal lymphoma (2)	Squamous cell carcinoma of skin	Acute lymphocytic leukemia	
Lung cancer		Seminoma	Basal cell carcinoma		
Gastric cancer		Cutaneous T‐cell lymphoma	Plasma cell myeloma		
Esophageal carcinoma		Acute leukemia	Acute myeloid leukemia (2)		
Prostate cancer (2)		Angioimmunoblastic T‐cell lymphoma	Gallbladder adenocarcinoma		
Tongue neoplasm malignant		Rectal cancer	Cervix carcinoma		
		Prostate cancer (2)	Ovarian cancer		
		Mantle cell lymphoma			
		Skin cancer			
		Squamous cell carcinoma			
		Hodgkin's disease			
		Neoplasm of orbit			
		Malignant melanoma (2)			
		Thyroid cancer			
		Lung neoplasm			
		Colorectal cancer			
		Lymphogranuloma			
		Lung adenocarcinoma			

*Note*: In brackets, the number of cases is given.

EudraVigilance data on SARs related to IL‐23 inhibitor prescription show that different typologies of cancer are represented in patients with psoriasis compared to the total number of SARs related to drugs prescribed to these patients. Oncologic SARs were 9.96%, 16.4%, and 19.3% for guselkumab, risankizumab, and tildrakizumab, respectively, in comparison to the total number of serious SARs sent for these three drugs (Table 2). Table 2 reports the results of the disproportionality test, calculated as the odds ratio comparing each drug with the other two IL‐23 inhibitors. The results indicate that the risk of suspected oncological adverse reactions to risankizumab and tildrakizumab is >1, compared to the same risk for the other two IL‐23 inhibitors. This is not observed for guselkumab, which shows a minor risk compared to the other two drugs.

**TABLE 2:** Reporting odds ratio (ROR) values for oncologic suspected adverse reactions (SARs) related to the prescription of IL‐23 inhibitors guselkumab, risankizumab, and tildrakizumab in psoriatic patients of the European Economic Area and the United Kingdom in the years 2020–2021–2022.

Drug	Oncologic SARs Total, *N* = 53	Percentage of oncologic SARs compared to all SARs for each IL‐23 inhibitor (%)	ROR (95% CI) calculated against the other two IL‐23 inhibitors
Guselkumab	28	9.96	0.49 (0.27–0.89)
Risankizumab	19	16.4	1.81 (0.98–3.34)
Tildrakizumab	6	19.3	1.66 (0.66–4.25)

Abbreviations: CI, confidence interval; IL, interleukin.

The disproportionality test was also applied to analyze the sex distribution of SARs among patients with psoriasis taking three different IL‐23 inhibitors. Data show that the risk of suspected oncologic adverse reactions related to risankizumab and tildrakizumab is >1 (2.3 and 2.4, respectively) for men. The risk of risankizumab use is >1 (1.15) in women. No risk was observed with guselkumab administration (Table 3).

**TABLE 3:** Reporting odds ratio (ROR) values and gender distribution of oncologic suspected adverse reactions (SARs) related to the prescription of IL‐23 inhibitors guselkumab, risankizumab, and tildrakizumab in the European Economic Area and the United Kingdom in the years 2020–2021–2022.

Drug	Oncologic SARs in women *N* = 19	ROR (95% CI) calculated against the other two IL‐23 inhibitors in women	Oncologic SARs in men *N* = 34	ROR (95% CI) calculated against the other two IL‐23 inhibitors in men
Guselkumab	12	0.86 (0.17–0.75)	16	0.36 (0.32–2.3)
Risankizumab	5	1.15 (0.39–3.40)	14	2.3 (1.07–4.86)
Tildrakizumab	2	1.10 (0.23–5.26)	4	2.4 (0.72–8.02)

Abbreviations: CI, confidence interval; IL, interleukin.

## 4 DISCUSSION

The results of the disproportionality test, calculated as the odds ratio obtained by comparing each IL‐23 inhibitor with the other two drugs belonging to the same class, suggest that when they are prescribed in patients with psoriasis, the risk for suspected oncologic adverse reactions is >1 for the drugs risankizumab (ROR = 1.81) and tildrakizumab (ROR = 1.66), whereas data on guselkumab do not confirm this view. Furthermore, data obtained through analysis performed after sex distribution indicated a difference in the risk of oncological SARs between men and women, which was greater in men for both risankizumab (ROR = 2.3) and tildrakizumab (ROR = 2.4). However, disproportionality analysis performed using a pharmacovigilance database requires support from a pharmacodynamic hypothesis established on the basic properties of drugs.

Regarding the biological plausibility of the possible link between IL‐23 inhibitor therapy and the potential occurrence of oncological adverse events, scientific literature does not present conclusive evidence. However, IL‐23 plays a key role in T‐cell–mediated immunity and the pro‐inflammatory reaction associated with autoimmune conditions. IL‐12 and IL‐23 may play defined roles in the immune response against tumors and bacterial infections. Thus, it has been proposed that therapies targeted to IL‐12 and IL‐23 carry a theoretical risk of reduced defenses versus pathogens and tumor surveillance. It is noteworthy that an enhanced risk of cancer has been detected among patients with psoriasis compared to the general population and that therapies with IL‐23 as a target represent a theoretical risk of reduced defense against tumors. IL‐23 is a cytokine that promotes carcinogenesis, and its levels are correlated with low prognosis in human cancer. Furthermore, large‐scale genome‐wide association research has identified single‐nucleotide polymorphisms in IL‐23 as elements that increase the risk of malignancy. Moreover, concerns regarding the enhanced risk of cancer have been raised with other immunosuppressive treatments for psoriasis, such as anti–TNF inhibitors, due to the role of TNF in cancer growth inhibition.^11^

However, a study involving the analysis of long‐term phase I–III clinical trials on patients with psoriasis indicated that the IL‐23 inhibitor risankizumab did not increase the risk of malignancies over those already associated with the pathophysiology of psoriasis.^12^ Moreover, the results of a meta‐analysis including 48 studies published until June 2020 investigating the clinical effects of IL‐23 inhibitors did not reveal any relationship between their use and cancer incidence.^13^ A suspected higher risk of cancer in male patients with psoriasis was noted in our study and in previous reports; however, only a few studies have discussed the sex distribution of cancer risk in patients with psoriasis. The association between psoriasis and malignancy was explored in a retrospective cohort study in the Taiwan National Health Insurance Research Database, including 3686 patients with diagnosis of psoriasis. The results showed that malignancies risk was major in male than in female patients (hazard ratio 1.86 vs. 1.14, respectively).^14^ Based on this evidence, a possible explanation for the difference observed in the EudraVigilance data in the sex distribution of oncologic SARs to IL‐23 inhibitors can be supported by the pre‐existing higher prevalence of cancer in male patients with psoriasis.

In conclusion, the limitations of the present study are typical of large databases containing real‐world data, which have strengths in terms of the number of reports, and weaknesses in terms of the poor quality of single reports. Other problems that reduce the strength of these data could be represented by the so‐called Weber effect (a peak in adverse reaction reporting relating to relatively new drugs), as it occurs for all drugs marketed recently, or the notoriety effect (selection bias detectable when a case shows a higher chance of being reported if the patient is exposed to the investigated factor causing, thought to cause, or probably to cause an event of interest). In addition, this type of post‐marketing surveillance lacks a denominator and shows incomplete reporting, which consequently reduces the value of the comparison among IL‐23 inhibitors, as reporting rates cannot be considered to reflect incidence rates. In this case, since guselkumab was first approved, it may be that non‐oncologic SARs are more commonly reported than the newer IL‐23 blockers. Furthermore, the database contains single signals as SARs, meaning any adverse event for which there is a reasonable possibility but not certainty that the drug could have caused it. Nevertheless, although randomized placebo‐controlled studies are generally regarded as the most reliable way to assess risk, the data from the present study, although indicative, seem to confirm the potential association between the IL‐23 inhibitors risankizumab and tildrakizumab and the occurrence of SARs linked to different types of cancer in patients with psoriasis, and show that this relationship is asymmetrically distributed between women and men according to a gender perspective, with a clear prevalence of oncologic SARs in men.

## AUTHOR CONTRIBUTIONS

All the authors provided their contribution as follows: conceptualization, Fabrizio Calapai, Ilaria Ammendolia, Carmen Mannucci; methodology, Mariaconcetta Currò; software, Ilaria Ammendolia, Luigi Cardia; validation, Gioacchino Calapai, Emanuela Esposito and Luigi Cardia; formal analysis, Fabrizio Calapai; data curation, Ilaria Ammendolia, Mariaconcetta Currò, Luigi Cardia; writing—original draft preparation, Fabrizio Calapai, Ilaria Ammendolia, Mariaconcetta Currò, Luigi Cardia; writing—review and editing, Gioacchino Calapai, Carmen Mannucci, Emanuela Esposito; supervision, Gioacchino Calapai; project administration, Fabrizio Calapai.

## CONFLICT OF INTEREST STATEMENT

The authors declare no conflict of interest.

## ETHICS STATEMENT

The authors signed an authorship responsibility form.

## Data Availability

The data that support the findings of this study are openly available in the following public domain resource https://www.ema.europa.eu/en/human‐regulatory/research‐development/pharmacovigilance/eudravigilance.
